# Characterization of Fresh Pasta Made of Common and High-Amylose Wheat Flour Mixtures

**DOI:** 10.3390/foods11162510

**Published:** 2022-08-19

**Authors:** Alessio Cimini, Alessandro Poliziani, Gabriele Antonelli, Francesco Sestili, Domenico Lafiandra, Mauro Moresi

**Affiliations:** 1 Dipartimento per l’Innovazione nei Sistemi Biologici, Agroalimentari e Forestali, Università della Tuscia, Via S. C. de Lellis, 01100 Viterbo, Italy; 2 Dipartimento di Scienze Agrarie e Forestali, Università della Tuscia, Via S. C. de Lellis, 01100 Viterbo, Italy

**Keywords:** amylose, consistency, energy consumption, cooking loss, egg-free fresh pasta, in vitro glycemic index, resistant starch

## Abstract

This study aims to assess the main biochemical, technological, and nutritional properties of a few samples of fresh pasta composed of commercial common wheat flour blended with increasing percentages, ranging from 0 to 100%, of high-amylose wheat flour. Although the technological parameters of such samples remained practically constant, fresh pasta samples including 50 to 100% of high-amylose wheat flour were classifiable as foods with a low in vitro glycemic index of about 43%. However, only fresh pasta made of 100% high-amylose wheat flour exhibited a resistant starch-to-total starch ratio greater than 14% and was therefore eligible to claim a physiological effect of improved glucose metabolism after a meal, as according to EU Regulation 432/2012.

## 1. Introduction

Increasing the amount of amylose in wheat grain is a target of breeding programs as it improves the nutritional profile of wheat by increasing its fraction of resistant starch (RS), which is undigested in the small intestine by amylases and fermented only by intestinal microbiota; this improves glucose metabolism after a meal [1]. Starch is known to be a mixture of two glucan homopolymers, amylose and amylopectin, that have the same backbone but differ in the degree of polymerization and ramification [2]. Amylose is smaller and mainly linear compared to amylopectin, which is highly branched. Although the amylose–amylopectin ratio is 1:3 in normal wheat grain, genotypes with an amylose content higher than 70% of the total starch content were obtained in bread wheat [3,4,5], giving rise to flours with RS content greater than 10%. These have been used to produce foods enriched in resistant starch, such as dried noodles [6], Japanese noodles, bread, and puffed grains [7]. By resorting to differential scanning calorimetry, X-ray diffraction, and polarized light microscopy, Li et al. [8] succeeded in monitoring the formation of a more retained crystalline structure, a lower loaf volume, a denser crumb structure, and higher hardness in high-amylose wheat bread than in conventional bread. A 50/50 mixture of control flour and high-amylose wheat flour produced bread that had not only one-fifth of the conventional bread digestion rate and about six times higher of an RS level while maintaining bread texture, but also required no modification in breadmaking conditions in terms of water addition and processing method. The slower pancreatic digestion and fermentability of high-amylose starch (HAS), and thus its potential physiological benefits along the gastrointestinal tract, were further confirmed by assessing via size-exclusion chromatography that the molecular structures of HAS unfermented residues were not altered during in vitro fermentation, while those of conventional starch exhibited a preferential degradation of the amylopectin fraction [9].

In vivo studies have demonstrated that high-amylose flours can be successfully used as raw material for producing low-glycemic-index (GI) foods. Vetrani et al. [10] provided rusks made with amylose-rich flour to overweight individuals and concluded that high-amylose rusks improved postprandial glucose metabolism and reduced the desire to eat, thus possibly contributing to the prevention of diet-related diseases such as obesity and diabetes. Sissons et al. [11] found similar results using high-amylose spaghetti, suggesting that a minimum RS content of 7% is needed in spaghetti to lower the GI.

It is useful to note that the European Regulation 432/2012 [12] allows functional foods with a RS level of at least 14% of their total starch content to be labeled with a specific health claim regarding the physiological effect of improved post-prandial glucose metabolism.

According to Italian Presidential Decree no. 187/2001 [13], durum wheat (*Triticum durum*), common wheat (*Triticum aestivum*), or their blends can be used for fresh pasta production, while dried pasta must be made with durum wheat semolina only.

The primary aim of this work is to assess the main biochemical (i.e., total starch, resistant starch), technological (e.g., optimal cooking time, water absorbed by cooked pasta, cooking loss, cooked pasta texture properties, cooking energy), and nutritional (that is, in vitro glycemic index) properties of some samples of egg-free fresh pasta (spaghetti), where a commercial common wheat flour type 00 was substituted with increasing fractions (x_F_) of a high-amylose wheat flour in the range of 0 to 100% (*w*/*w*).

## 2. Materials and Methods

### 2.1. Raw Materials

A derived high-amylose line of the bread wheat cultivar Cadenza [3] was grown in an open field at the Massimo Filzi Agricultural Farm located in Anguillara Sabazia (Rome, Italy: latitude 42.060215 and longitude 12.258220 expressed in decimal degrees, altitude 208.97 m a.s.l.) during the season of 2019–2020. Crop management was performed using standard cultivation methods and direct drilling, by placing the seed in the stubble of the previous crop without any prior soil cultivation. Nitrogen fertilization was split into three applications: the first application was given before sowing, as diammonium phosphate (200 kg/ha); the second one when the first node was detectable above ground, as urea (150 kg/ha); and the third one 25 days later, as ammonium nitrate (150 kg/ha). About 162 kg of grain was ground in a pilot-scale mill by Grandi Molini Italiani SpA (Venice, Italy: www.grandimolini.it (accessed on 10 August 2020), thus obtaining 91 kg of high-amylose wheat flour (HAWF). A common wheat flour type 00 (00 BWF) was provided by Molino Profili Giuseppe sas (Viterbo, Italy). The flour was characterized by a dough strength (W) of 180–200 (10^−4^ J), and an alveograph ratio (P/L) between the maximum pressure (P in mm) and extensibility (L in mm) of 0.5–0.6.

### 2.2. Fresh Pasta Production

Several flour mixtures with a weight fraction (x_F_) of HAWF in the range of 0 to 20, 50, 70, or 100% *w*/*w* were prepared using pre-weighted amounts of both flours, which were then carefully mixed to assure homogeneity.

Kneading was carried out in a batch mixer of type Simac Pastamatic 1000 (De’ Longhi Appliances Srl, Campi Bisenzio, FI, Italy) for 8 min by adding deionized water at a rate of 35 g for every 100 g of flour for x_F_s ranging from 0 to 0.7 g/g. At x_F_ = 1 g/g the hydration rate was increased to 37 g/100 g. The resulting dough was drawn in the form of fresh spaghetti, which was stored inside appropriately closed aluminum trays at +4 °C for not longer than two days. Each fresh pasta sample was replicated three times using the same lot of flour mixture. The optimal cooking time (OCT) was determined according to the ISO 7304–1 method [14].

### 2.3. Fresh Pasta Cooking

Cooking was carried out using a previously developed eco-sustainable cooker [15]. This cooker consisted of the following items: (i) a 2-kW induction-plate hob (Melchioni INDU, Melchioni Spa, Milan, Italy), (ii) a 3-L induction stainless steel cooking pot (model Oumbärlig, Inter IKEA Systems B.V., Delft, Netherlands), (iii) a stainless-steel rod mixer piloted by a 12-V direct-current electric motor welded to the pot lid, (iv) a digital temperature sensor to monitor the cooking water temperature, and (v) a current sensor to register the electric energy consumption. The operation of this eco-sustainable cooker was handled via an Arduino^®^ hardware platform and an application installed on a smartphone with an Android operating system [15].

All cooking tests were performed using 50 g of each fresh pasta sample in 150 or 500 g of bottled still mineral water with a fixed residue at 180 °C of about 300 mg/L [14] to set the water-to-fresh-pasta ratio (WPR) as 3 or 10 L/kg, respectively. As soon as the pot was filled with the water amounts mentioned above and closed with a lid, the induction hob was set at its maximum power rating (2 kW) to not only heat the cooking water from ~15 to 98 °C, but also reheat the water-pasta mixture to 98 °C after pasta pouring once the lid had been reclosed. Thereafter, the hob power rating was automatically lowered to its minimum value (i.e., 200 W). To avoid spaghetti sticking, the stirrer mentioned above was automatically activated 30 s after the addition of fresh pasta, and kept rotating at 30 rev/min for 10 s, after which it was rested for the subsequent 20 s. During the pasta cooking phase, the microprocessor system acquired the water temperature every 10 s, and automatically increased or decreased the power rating in steps of 200 W depending on whether the current cooking water temperature was lower or higher than 98 °C, respectively. The pasta cooking process was prolonged up to the corresponding optimal cooking time (OCT). The electricity consumed by the induction hob (E_S_) was also measured with a digital power meter type RCE MP600 (RCE Srl, Salerno, Italy).

### 2.4. Cooked Pasta Analyses

Cooked pasta pieces were recovered from the pasta water using a colander and cooled according to Method 66–50.01 of the American Association of Cereal Chemists [16]. Such a method was also used to assess the water uptake by cooked pasta (WU), as well as the solids dispersed in cooking water (cooking loss, CL), both parameters being related to a unitary mass of fresh pasta [17].

The textural properties of cooked pasta were assessed using a universal testing machine of UTM mod. 3342 (Instron Int. Ltd., High Wycombe, UK), equipped with a 1000 N load cell. As described previously [18,19], 17 strands of cooked spaghetti were aligned over a stainless-steel platen and tested using a trapezoidal cutting probe. The average diameter (Ø) of cooked spaghetti was calculated as the difference between the total displacement of the probe at the contact point with the platen and sample, as revealed by a force of 0.05 N. Each test was carried out by setting the probe speed at 1 mm/s. A first bite was performed by submitting the strands to a 30% compression. Then, the probe was raised to its starting position. After a short relaxation time of 5 s, it was newly lowered to submit the specimens to a second 90% compression and then lifted to its initial position. According to Bourne [20], the force peak on the first or second compression cycle was defined as the pasta hardness at 30% (F_30_) or 90% (F_90_) deformation, while the ratio between the force-versus-time area (AC_90_) during the second compression and the force-versus-time area (AC_30_) during the first compression was defined as *cohesiveness* or *cohesion energy resilience* (CER). The distance at which the strands recovered their height during the time that elapsed between the end of the first bite and the start of the second bite was defined as *springiness* (S). Each test was repeated five times.

Total starch (x_TS_) and resistant starch (x_RS_) fractions in flours and cooked pasta samples were determined using the total starch (amyloglucosidase/α-amylase method) and resistant starch kits by Megazyme Ltd. (Bray, Ireland), respectively. Fresh pasta samples were cooked for as long as the OCT and directly tested in the case of RS, while they were dried and then ground for the total starch assessment.

### 2.5. Water Balance and Energy Efficiency of Fresh Pasta Cooking

The mass of the cooking water was measured at the beginning (*m_W_*_0_) of the cooking process, while that of the pasta water (*m_WF_*) was determined once the cooked pasta was recovered. Then, the water uptake by cooked pasta (*m_WPA_*) was calculated by subtracting the mass of the cooked pasta (*m_CP_*) from that of fresh raw pasta (*m_PA_*), while the water evaporated (*m_WE_*) throughout the cooking process was calculated as follows:

*m_WE_* = *m_W0_* − *m_WPA_* − *m_WF_* (1)

In this way, it was possible to calculate the fractions of cooking water evaporated (*η_WE_)*, adsorbed by cooked pasta (*η_WPA_*), or recovered as pasta water (*η_WF_*).

The energy efficiency (*η_C_*) of the pasta cooking system was estimated by dividing the sensible heat needed to raise the initial temperatures of the cooking water (*T_W_*_0_) and fresh pasta (*T_PA_*_0_*)* to the boiling point of water (i.e., *T_B_* ≅ 98 °C) by the energy supplied by the induction hob (*E_S_)*:

(2) $$\eta_{C}=\frac{m_{W0}c_{pW}\left(T_{B}-T_{W0}\right)+m_{PA}c_{pPA}\left(T_{B}-T_{PA0}\right)}{E_{S}}$$

where *c_pW_* and *c_pPA_* are the specific heat of water (=4.186 kJ kg^−1^ K^−1^) and fresh raw pasta, respectively. As the x_F_ was increased from 0 to 1 g/g, the moisture content of fresh pasta samples increased from 0.352 to 0.374 g/g, this making the specific heat values increase from 2.45 to 2.51 kJ kg^−1^ K^−1^, according to the data derived from Singh et al. [21].

### 2.6. In Vitro Starch Digestion and Glycemic Index

In vitro digestion of pasta starch was determined according to the method developed by Zou et al. [22]. All the tests were replicated at least 3 times. The concentration of glucose released (C_G_) from simulating the digestion of starch in the mouth and stomach through appropriate enzymatic treatments was determined using an enzymatic kit (D-Glucose Assay Procedure, K-GLUC 07/11, Megazyme Ltd., Bray, Ireland), at different times (*t*) ranging from 0 to 360 min. The area under the C_G_-*versus*-t curve (digestogram) for a digestion time ranging from 0 to 180 min (*AUC*) for each fresh pasta sample was normalized with respect to the corresponding area for a reference product (i.e., white bread, its total starch content being 83.4 ± 1.7 g/g dm), as suggested by Giuberti et al. [23]. Such a ratio was defined as the *starch hydrolysis index* (*SHI*), which was expressed as a percentage.

The area under the digestogram (*AUC*) up to an overall incubation time (*t_f_)* of 180 min is expressed as

(3) $$AUC=\int_{0}^{t_{f}}C_{G}\left(t\right)dt$$

where *t* is the digestion time and *C_G_(t)* is the instantaneous concentration of glucose released. The value of such a definite integral can be calculated either numerically according to the trapezoidal rule or analytically by expressing *C_G_(t)* via the first-order exponential model previously employed by Goňi et al. [24] and modified by Mahasukhonthachat et al. [25]:

*C_G_(t)* = *C_G_*_0_ + (*C_G__∞_* − *C_G_*_0_) (1 − *e^−k^ ^t^*) (4)

where *C_G_*_0_ is the initial concentration of glucose freed at time *t* = 0 as resulting from the very rapid digestion of starch (both in the mouth and stomach as simulated using artificial saliva and pepsin treatments), *C_∞_* is the concentration of glucose potentially released for *t_∞_*, and *k* is the digestion rate constant.

By introducing Equation (4) into Equation (3), the above definite integral can be analytically solved as follows:

(5) $$AUCE\;=C_{G\infty }\;t_{f}-\frac{C_{G\infty }-C_{G0}}{k}\left(1-e^{-k\;t_{f}}\right)$$

For the purpose of data fitting, *C_G__∞_* and *k* values can be obtained by

- Setting an initial iterative value for *C_G__∞_*;
- Linearizing Equation (4) as follows:
  *ln* {[*C_G__∞_* − *C_G_(t)*]/(*C_G__∞_* − *C_G_*_0_)} = −*k t*, (6)
- Determining *k* via the least squares method.
- Minimizing the mean percentage error among the experimental and calculated *C_G_* values with respect to *C_G__∞_*, using a non-linear estimation method.

Alternatively, the glucose release kinetics are well described by the Peleg model [25], despite the fact that this model was originally used to simulate moisture sorption curves [26]. Such a model was re-written in terms of released glucose as

(7) $$C_{G}\left(t\right)=C_{G0}+\frac{t}{k_{1}+k_{2}\;t}$$

where *k*_1_ is the Peleg rate constant, and *k*_2_ the Peleg capacity constant. The initial rate of glucose release is

(8) $${\frac{dC_{G}}{dt}]}_{t=0}=\frac{1}{k_{1}}$$

while at *t→∞*, the glucose potentially released becomes

(9) $$C_{G\infty }=\;C_{G0}+\frac{1}{k_{2}\;}$$

Thus, the initial glucose release rate is inversely proportional to the Peleg rate constant *k*_1_, while the Peleg capacity constant *k*_2_ is related to the equilibrium glucose released (*C_G__∞_*). The area under the digestogram can be analytically calculated as follows:

(10) $$AUC_{Peleg}=\int_{0}^{t_{f}}C_{G}\left(t\right)dt=C_{G0\;}t_{f}+\frac{t_{f}}{k_{2}}-\frac{k_{1}}{k_{2}^{2}}\;\ln\left(\frac{k_{1}+k_{2}\;t_{f}}{k_{1}}\right)$$

The Peleg constants *k*_1_ and *k*_2_ can be simultaneously determined using the least squares method once Equation (7) is linearized as follows:

(11) $$\frac{t}{C_{G}\left(t\right)\;{-\;G}_{G0}}{=k}_{1}{+k}_{2}\;t,$$

Both the exponential and Peleg models are valuable to understand the extent and rate of glucose release from starch digestion in food products.

As the starch hydrolysis index *SHI* mentioned above was estimated, a predictive in vitro glycemic index (*GI*) was calculated for each fresh pasta sample using the empirical formula suggested by Granfeldt et al. [27]:

*GI* = 8.198 + 0.862 × *SHI* (12)

by assuming *SHI* of white bread as equal to 100.

### 2.7. Statistical Analysis of Data

All the tests were replicated at least 3 times to estimate the mean value and standard deviation of all parameters under examination. Statistically significant differences were analyzed using a Tukey test at a probability level (p) of 0.05. One-way analysis of variance (ANOVA) was also carried out using SYSTAT version 8.0 (SPSS Inc., Chicago, IL, USA, 1998).

## 3. Results and Discussion

### 3.1. Composition of Raw Materials

Table 1 shows the chemical composition of the high-amylose (HAWF) and type 00 (00 BWF) common wheat flours used in this work.

In HAWF, the amylose-to-total-starch ratio was greater than 80% (84.3 ± 0.3%), while the resistant starch-to-total starch-ratio (RS/TS) was equal to (17 ± 1) %. In principle, such a flour might be used to formulate functional foods that exert a physiological effect of improved post-prandial glucose metabolism; such a health claim would comply with EU Regulation no. 432/2012 [12], provided that the RS/TS ratio is at least 14%.

### 3.2. Composition of Fresh Pasta and Technological Parameters

**Figure 1:**
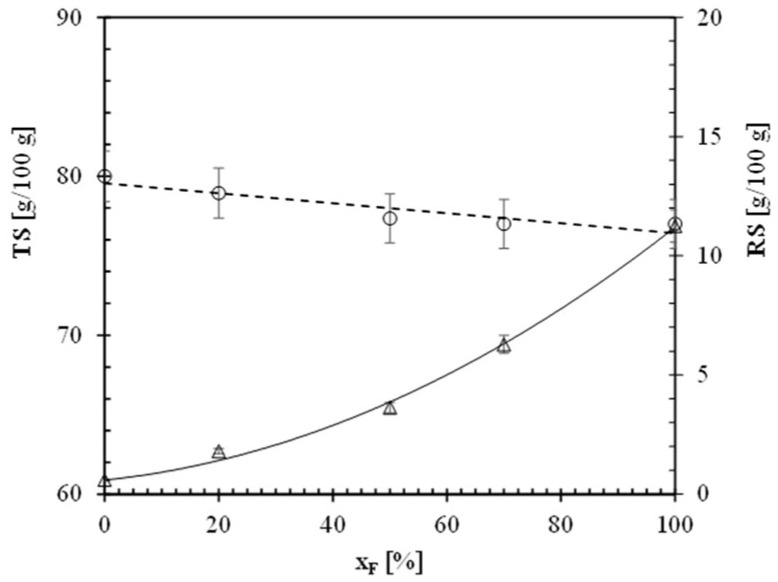
Effect of the percentage (x_F_) of high-amylose wheat flour in the flour mixture used to prepare fresh pasta samples on the total starch (x_TS_: ○) and resistant starch (x_RS_: △) weight fractions of the pasta.

Table 2 shows the chemical composition of fresh pasta samples made from a flour mixture consisting of 0 to 100% HAWF. As shown in Figure 1, their total starch content (x_TS_) on a moisture-free basis exhibited a linear decrease (r^2^ = 0.97) from 80% to 77% as the x_F_ increased from 0 to 1.0 g/g. In this x_F_ range, the fraction of resistant starch (x_RS_), that is, the fraction of starch that is not hydrolyzed in the small intestine and is partially or totally fermented in the large intestine [28], exhibited an upwardly curved trend (R^2^ = 0.997) from 0.59 to 11.3% in the cooked fresh pasta samples.

By carrying out the cooking tests with a water-to-fresh-pasta ratio (WPR) of 10 L/kg, the time at which the central white core of the fresh pasta strands under study appeared as broken (OCT) or fully disappeared reduced from 3.5 to 3 min as the x_F_ increased from 0 to 100%. Similar OCT values were obtained at WPR = 3 L/kg (Table 2). As previously observed [29], such data exhibits a standard deviation up to about 30 s and therefore are not statically different at the 95% confidence level. Whatever the WPR used, the OCT for all samples tested can in practice be regarded as constant.

Table 2 also shows that the relative water uptake (WU) of fresh spaghetti samples cooked near or at the water boiling point (~98 °C) using two WPRs may be regarded as approximately constant, the differences among the experimental data being not statistically significant at the probability level (p) of 0.05. Thus, the average value of water uptake per g of fresh pasta (0.73 ± 0.06 g/g) can be considered as independent not only of the HAWF content but also of the WPR. When all fresh pasta samples were cooked in an excess of water (WPR = 10 L/kg), the cooking loss was practically independent of the x_F_ and equal to 0.075 ± 0.012 g/g, which did not differ from that detected by Sissons et al. [11] when dry high-amylose durum wheat spaghetti was cooked with the same WPR. When the fresh spaghetti samples were cooked at WPR = 3 L/kg, their strands tended to agglomerate slightly, thus lowering the surface area of pasta directly contacting the cooking water. Consequently, the solid matter dissolution rate reduced to 0.056 ± 0.012 g/g, as also observed previously [29,30].

### 3.3. Textural Properties of Cooked Pasta

Despite some slight statistically significant differences (see Table 3), the main textural properties of the cooked spaghetti samples appeared to be practically indifferent of both x_F_ and WPR. Altogether, the cooked samples exhibited almost the same diameter (Ø = 2.31 ± 0.07 mm), hardness at both 30% (F_30_ = 3.5 ± 0.5 N) and 90% (F_90_ = 11.9 ± 1.6 N) deformation, springiness (S = 2.06 ± 0.07 mm), and cohesiveness (CER = 14.2 ± 3.9).

**Figure 2:**
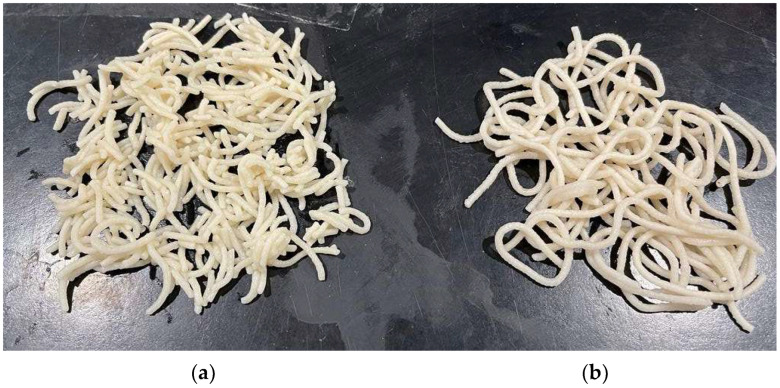
Cooked fresh pasta samples made of common and high-amylose wheat flours, the percentage fraction (x_F_) of the latter being equal to 1 (**a**) or 0.7 (**b**) g/g.

It is well known that the above texture parameters are highly correlated to sensory ratings [20]. More specifically, cooked pasta hardness is associated with the sensory attributes of firmness, stickiness, bulkiness, and overall cooking quality of cooked pasta [31], this finding having been also confirmed in previous work [19]. Thus, the results shown in Table 3 reveal no statistically significant difference in the textural properties of the cooked fresh pasta samples tested here, even if the fresh pasta samples made of HAWF only exhibited a lower hardness at 90% compression (2nd bite), as well as a greater tendency to break down during cooking, than the other samples at x_F_s ranging from 0 to 0.7 g/g (Figure 2).

### 3.4. Cooking Energy Needs and Water Use

Table 4 lists the results of the cooking tests performed under the two water-to-pasta ratios mentioned above. 

When using 10 L of water to cook 1 kg of fresh pasta prepared using 0–0.7 g of HAWF in the flour mixture, the specific electric energy supplied (*E_S_*) during the water heating and pasta cooking phase was approximately constant (2.0 ± 0.3 kWh/kg). Similarly, the utilization of cooking water was nearly stable, with the percentages of water evaporated (*η_EV_*), absorbed by cooked pasta (*η_CP_*) or remaining as pasta water (*η_PW_*) being equal to 9.0 ± 1.3%, 7.0 ± 0.4%, or 84.0 ± 1.6% of the water initially added into the pot (*m_W_*_0_), respectively. 

By reducing WPR to 3 L/kg, *E_S_* appeared to be independent of the HAWF percentage in the flour mixture (x_F_ = 0–0.7 g/g) but reduced to 1.08 ± 0.06 kWh/kg.

Similarly, the cooking water utilization was almost steady, with the percentages of water released as steam (*η_EV_*) and absorbed by cooked pasta (*η_CP_*) increasing to 29 ± 5% and 24 ± 2% of the m_W0_, respectively. Even if the percentage of pasta water (*η_PW_*) reduced to 47 ± 6% of the *m_W0_*, no shortage of cooking water was accounted for, this being indirectly corroborated by the invariance in cooked pasta hardness as deducible from the Tukey test shown in Table 3.

By using Equation (2), the overall cooking energy efficiency (*η_C_*) for each fresh pasta sample was found to be about constant (51 ± 5%) at a WPR of 10 L/kg but reduced to 32 ± 2% at a WPR of 3 L/kg, owing to the greater fraction of water evaporated (*η_WE_*) (Table 4). Such cooking energy efficiencies, which refer to cooking tests performed with just 50 g of fresh pasta, were smaller than those (i.e., *η_C_* = 71 ± 4% at WPR = 10 L/kg, and *η_C_* = 52 ± 6% at WPR = 3 L/kg) previously observed when cooking 250 g of dry durum wheat semolina pasta (see Table S1 in the supplementary material in the online version of ref. [15]).

In all probability, the amount of cooking water used (i.e., 500 or 150 g), as contained in the same 3-L induction stainless steel cooking pot used in previous trials, created a greater surface-to-volume ratio of water exposed to the environment that resulted in a greater fraction (*η_EV_*) of water evaporated. To sustain such a hypothesis, the amount of fresh pasta samples at x_F_ = 1 g/g was increased to 150 g. In such cooking tests, at WPR = 10 L/kg the fraction of water evaporated reduced from (9.0 ± 1.3) % to (2.8 ± 0.2) %, while at WPR ≅ 3 L/kg *η_EV_* reduced from (24 ± 2) % to (16 ± 1) % (Table 4). In these circumstances, the *η_C_* increased to 80 ± 2% at WPR = 10 L/kg, and to 65 ± 2% at WPR = 3 L/kg, these values being in line with those previously observed [15]. Finally, at the low cooking water-to-pasta ratio of 3 L/kg, the fraction of water absorbed by cooked fresh pasta (*η_CP_*) appeared to be independent of the amount of fresh pasta undergoing cooking (Table 4).

Further energy savings might be achieved by cooking pasta at lower temperatures than the water boiling point, as previously observed [29].

### 3.5. In Vitro Starch Digestion and Glycemic Index

**Figure 3:**
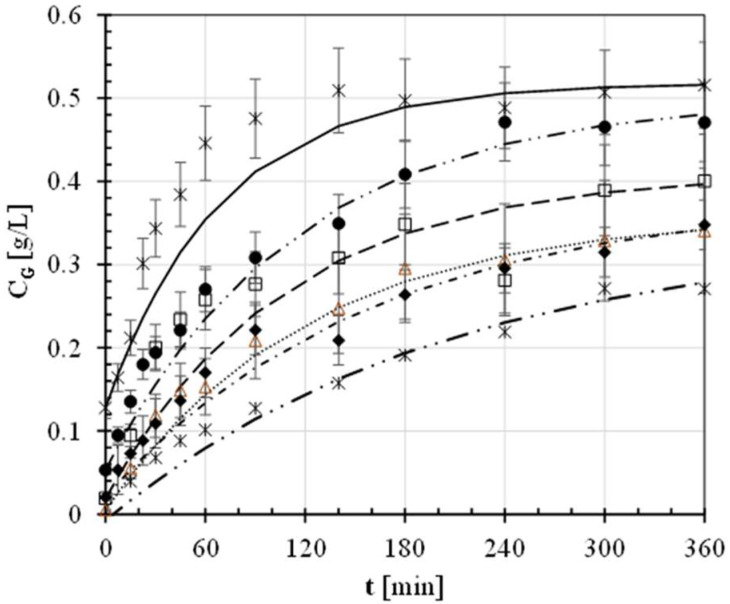
Time course of simulated in vitro starch digestion using white bread (✕, —) or cooked fresh pasta samples enriched with different percentages (x_F_) of high-amylose wheat flour (⬤, — ⋅ ⋅ —: x_F_ = 0 g/g; ☐, - - -: x_F_ = 0.2; g/g; △, ……: x_F_ = 0.5 g/g; ◆, — ⋅ —: x_F_ = 0.7 g/g; ∗, — ⋅ ⋅ —: x_F_ = 1 g/g): comparison between the experimental concentrations of glucose released (C_G_: open or closed symbols) and those calculated (continuous, broken or dash-dotted lines) using the exponential model (Equation (4)) and corresponding empirical constants listed in Table 5 vs. time (t).

To describe the starch digestion kinetics in the mouth and stomach through appropriate enzymatic treatments for the samples tested, the average concentration (*C_G_*) of experimentally freed glucose was plotted as a function of the incubation time (*t*), as shown in Figure 3.

The time course of *C_G_* was firstly described by using the exponential model (Equation (4)) to determine the empirical constants shown in Table 5. The continuous, broken, and dash-dotted lines plotted in Figure 2 allow for quite an accurate reconstruction of the experimentally observed digestograms. From Table 5 it can be noted that the digestion rate constant (*k*) for each of the cooked fresh pasta samples was significantly smaller than that pertaining to white bread at *p* = 0.05 and tended to reduce as the addition of HAWF was increased. However, the constants relative to the samples enriched with 0 to 0.5 g/g of HAWF were approximately constant at the probability level of 0.05 and definitively greater than the digestion rate constant for the cooked samples comprised of 70% HAWF.

The area under each digestogram up to an overall incubation time of 180 min (*AUC_E_*) was analytically calculated using Equation (5) and is shown in Table 5. It reduced from about 68 g min/L in the case of white bread to ~30 g min/L for the samples with x_F_ = 0.7 g/g (Table 5), even if the latter was not statistically different from the definite integral relative to the samples with x_F_ = 0.5 g/g.

The time course of C_G_ was also fitted using the Peleg model (Equation (7)) by estimating its empirical constants k_1_ and k_2_ (Table 5). The smaller value of the Peleg rate constant k_1_ was associated with white bread. Such a constant apparently increased with x_F_, this clearly indicating that the higher the HAWF content, the smaller the initial glucose release rate. Moreover, the k_1_ values relative to the fresh pasta samples containing 50 and 70% of HAWF were found to not be significantly different at *p* = 0.05.

The Peleg capacity constant k_2_, being inversely proportional to the difference between the equilibrium and initial glucose concentrations, was nearly constant (1.9 L/g) for the white bread and fresh pasta made of bread wheat flour only (x_F_ = 0 g/g), but it increased to ~2.5 L/g for the fresh pasta samples containing from 0.2 to 0.7 g/g of HAWF (Table 5). Thus, the higher the resistant starch content in fresh pasta, the lower the equilibrium glucose released by starch digestion. The area under each digestogram was then analytically calculated using Equation (10) and is listed in Table 5.

Despite the above mathematical models giving rise to a diverse fitting of the experimental C_G_ data (see for instance Figure 2 for t < 75 min), the estimated areas under the digestograms exhibited a noticeable agreement. Nevertheless, the trapezoidal rule was applied to obtain a more accurate calculation of the area under the digestograms (Table 5). AUC_Trap_ was equal to (77 ± 7) g min/L in the case of white bread but reduced to (51 ± 2) g min/L in the case of fresh pasta made of bread wheat flour only (x_F_ = 0 g/g). This result was firstly pointed out by Jenkins et al. [32], who reported that the glycemic response of diabetic subjects following the consumption of spaghetti was reduced compared to white wheat bread. The higher the x_F_, the smaller the equilibrium glucose (C_G__∞_) released from the simulated digestion of cooked pasta became, presumably because of the greater amylose content (Table 1). Thus, AUC_Trap_ reduced to (27 ± 1) g min/L for x_F_ = 1.0 g/g.

The ratio of the AUC value for each sample to the corresponding area for the reference product (white bread) allowed the starch hydrolysis index (SHI) to be estimated. It significantly decreased (*p* < 0.05) from (66 ± 2)% for x_F_ = 0 g/g to (36 ± 1)% for x_F_ = 1.0 g/g. Additionally, the structure of pasta has been described as a compact matrix with starch granules entrapped in a protein network [33], this slowing starch digestibility in cooked pasta [34].

The in vitro glycemic index (GI) was finally estimated using Equation (12). The concept of GI was introduced to classify high-carbohydrate-containing foods according to their effect on post-prandial blood glucose content. Foster-Powell et al. [35] classified foods into three categories: low- (≤55), medium- (55–69), and high- (≥70) GI foods. According to this classification system, further confirmed by Atkinson et al. [36], the fresh pasta enriched with 50 to 100% high-amylose wheat flour exhibited practically the same in vitro glycemic index of circa 43% at a confidence level of 95%, and thus would fall within the range of low-in-vitro-GI foods; these foods induce a small increase in the post-meal level of glucose in the blood and therefore lower the long-term risk of type 2 diabetes mellitus on top of preventing obesity and metabolic risk factors such as coronary heart disease [37]. These results agree with previous studies carried out on other high-amylose foods, such as rusks [10], bread [8,38,39,40], pasta [11], noodles [6], and semolina pudding [41].

That being said, the resistant starch levels in the cooked fresh pastas containing 50%, 70%, or 100% HAWF were 4.7%, 8.2%, and 14.7% of the total starch, respectively. Thus, only the fresh pasta made of 100% high-amylose wheat flour might be able to claim a physiological effect of improved glucose metabolism after a meal, according to EU Regulation 432/2012 [12].

## 4. Conclusions

This study determined the main biochemical, technological, and nutritional properties of fresh pasta samples obtained from a commercial common wheat flour blended with increasing percentages of a high-amylose wheat flour in the range of 0 to 100% (*w*/*w*). Not only the technological parameters of these cooked samples (i.e., optimal cooking time, water uptake, and cooking loss) but also their textural properties (i.e., hardness at 30% and 90% deformation, springiness, and cohesiveness) appeared to be practically insensitive to the fraction of high-amylose wheat flour added. As the cooking-water-to-pasta ratio was reduced from 10 to 3 L/kg of fresh pasta, there was no statistically significant variation in the water uptake per g of fresh pasta; however, the solid matter dissolution rate reduced by about 75% and cooking energy needs practically halved, this contributing to significantly mitigate the greenhouse gas emissions associated with fresh pasta cooking. In the cooked fresh pasta samples, the total starch content on a moisture-free basis linearly decreased from 80% to 77%, while the resistant starch fraction increased from 0.59 to 11.3%. Thus, fresh pasta made with type 00 common wheat flour blended with 50–70% of high-amylose wheat flour slowed the post-prandial digestion of starch and reduced the in vitro glycemic index of the pasta to about 45%. Such addition was, however, insufficient to claim the physiological effect of improved glucose metabolism after a meal as according to EU Regulation 432/2012. We found that fresh pasta made of 100% high-amylose wheat flour was a low-in-vitro-GI food with a resistant starch level greater than 14% of the total starch.

However, further work is needed to compare the cradle-to-grave environmental profile of this innovative functional food to that of conventional egg-free fresh pasta.

## Figures and Tables

**Table 1:** Chemical compositions of the high-amylose (HAWF) and type 00 (00 BWF) common wheat flours used in this work.

Component	HAWF	00 BWF	Unit
Moisture	14.3 ± 0.1 ^a^	12.7 ± 0.1 ^b^	g/100 g wet matter
Total starch (TS)	64.5 ± 0.4 ^a^	73.0 ± 0.5 ^b^	g/100 g wet matter
Resistant starch (RS)	10.9 ± 0.7 ^a^	0.1 ± 0.3 ^b^	g/100 g wet matter
Amylose (AM)	54.4 ± 0.5	n.d.	g/100 g wet matter
Raw protein (6.25 × N)	10.8 ± 0.1 ^a^	11.5 ± 0.3 ^b^	g/100 g wet matter
Fat	2.1 ± 0.2 ^a^	2.0 ± 0.2 ^a^	g/100 g wet matter
Fiber	n.d.	2.5 ± 0.2	g/100 g wet matter
Ash	0.50 ± 0.00 ^a^	0.50 ± 0.00 ^a^	g/100 g wet matter

n.d.: not determined. Different lowercase letters indicate statistically significant differences among the mean values of the chemical composition of the fresh pasta samples differing in x_F_ at the probability level of 0.05.

**Table 2:** Effect of the percentage (x_F_) of high-amylose wheat flour (HAWF) in the flour mixture used to prepare fresh pasta samples on the main biochemical and technological properties of the pasta when cooked at different water-to-pasta ratios (WPR).

Parameter	Fresh Pasta Enriched with High-Amylose Wheat Flour					Unit
HAWF content, x_F_	0	0.2	0.5	0.7	1.0	g/g
Moisture content, x_W_	31.2 ± 0.5 ^a^	31.6 ± 0.3 ^a^	30.7 ± 0.9 ^a^	30.8 ± 0.8 ^a^	32.6 ± 0.8 ^a^	%
Total starch content, x_TS_	80.0 ± 1.2 ^a^	78.9 ± 0.2 ^a^	77.4 ± 1.6 ^b^	77.0 ± 1.4 ^b^	77.0 ± 1.1 ^b^	%
Resistant starch content, x_RS_	0.59 ± 0.01 ^a^	1.8 ± 0.2 ^b^	3.64 ± 0.02 ^c^	6.3 ± 0.2 ^d^	11.3 ± 0.3 ^e^	%
*WPR =* 10 L/kg						
Optimal cooking time, OCT	3.5 ± 0.3 ^a^	3.5 ± 0.3 ^a^	3.0 ± 0.3 ^a^	3.0 ± 0.3 ^a^	3.0 ± 0.2 ^a^	min
Water uptake, WU	0.74 ± 0.02 ^a^	0.70 ± 0.02 ^a^	0.68 ± 0.02 ^a^	0.67 ± 0.07 ^a^	0.80 ± 0.02 ^b^	g/g
Cooking loss, CL	0.070 ± 0.002 ^a,b^	0.058 ± 0.007 ^b^	0.077 ± 0.015 ^a^	0.087 ± 0.011 ^a^	0.086 ± 0.016 ^a^	g/g
*WPR =* 3 L/kg						
Optimal cooking time, OCT	3.5 ± 0.3 ^a^	3.5 ± 0.3 ^a^	3.0 ± 0.3 ^a^	3.0 ± 0.3 ^a^	3.0 ± 0.2 ^a^	min
Water uptake, WU	0.75 ± 0.05 ^a^	0.74 ± 0.12 ^a^	0.69 ± 0.08 ^a^	0.74 ± 0.04 ^a^	0.74 ± 0.04 ^a^	g/g
Cooking loss, CL	0.047 ± 0.006 ^c^	0.044 ± 0.001 ^c^	0.054 ± 0.005 ^b,c^	0.059 ± 0.002 ^b^	0.076 ± 0.009 ^a^	g/g

Different lowercase letters indicate statistically significant difference among the parameter means of the fresh pasta samples differing in x_F_ at the probability level of 0.05.

**Table 3:** Effect of the percentage (x_F_) of high-amylose wheat flour in the flour mixture used to prepare fresh pasta samples on the textural properties of cooked pasta strands when using different water-to-pasta ratios (WPR).

Parameter	Fresh Pasta Enriched with High-Amylose Wheat Flour					Unit
HAWF content, x_F_	0	0.2	0.5	0.7	1.0	g/g
*WPR =* 10 L/kg						
Hardness, F_30_	2.2 ± 0.8 ^b^	3.1 ± 0.1 ^a^	3.7 ± 0.4 ^a^	3.9 ± 0.1 ^a^	3.9 ± 0.6 ^a^	N
Hardness, F_90_	11.3 ± 1.4 ^a,b^	13.3 ± 1.0 ^a^	12.8 ± 1.1 ^a^	11.8 ± 0.7 ^a^	9.2 ± 2.3 ^b^	N
Springiness, S	2.18 ± 0.08 ^a^	2.12 ± 0.03 ^a,b^	2.05 ± 0.04 ^b,c^	1.96 ± 0.04 ^c^	2.05 ± 0.07 ^b,c^	mm
*Cohesiveness,* CER	12.1 ± 3.0 ^a^	14.3 ± 0.6 ^a^	11.8 ± 1.3 ^a^	11.9 ± 2.1 ^a^	11.6 ± 2.4 ^a^	-
Initial diameter, Ø	2.46 ± 0.07 ^a^	2.36 ± 0.02 ^a,b^	2.30 ± 0.04 ^b^	2.20 ± 0.05 ^c^	2.29 ± 0.07 ^b^	mm
*WPR =* 3 L/kg						
Hardness, F_30_	2.8 ± 0.2 ^b^	3.2 ± 0.3 ^b^	3.9 ± 0.1 ^a^	4.1 ± 0.5 ^a^	3.3 ± 0.1 ^b^	N
Hardness, F_90_	13.0 ± 0.6 ^a,b^	12.8 ± 1.7 ^a,b^	13.6 ± 0.5 ^a^	11.8 ± 0.7 ^b^	8.9 ± 0.5 ^c^	N
Springiness, S	2.12 ± 0.02 ^a^	2.07 ± 0.04 ^a,b^	2.04 ± 0.07 ^a,b,c^	1.96 ± 0.04 ^c^	1.97 ± 0.09 ^b,c^	mm
*Cohesiveness,* CER	14.8 ± 0.1 ^a^	12.8 ± 1.1 ^b^	11.8 ± 2.0 ^b^	11.9 ± 2.4 ^b^	14.6 ± 2.3 ^a,b^	-
Initial diameter, Ø	2.37 ± 0.02 ^a^	2.32 ± 0.06 ^a,b^	2.29 ± 0.07 ^a,b,c^	2.20 ± 0.05 ^c^	2.22 ± 0.09 ^b,c^	mm

Different lowercase letters indicate statistically significant differences among the parameter means of the fresh pasta samples differing in x_F_ at the probability level of 0.05.

**Table 4:** Effect of the percentage (x_F_) of high-amylose wheat flour in the flour mixture used to prepare fresh pasta samples on the cooking energy needs (E_S_) and distribution of cooking water when using different water-to-pasta ratios (WPR).

Parameter	Fresh Pasta Enriched with High-Amylose Wheat Flour					Unit
HAWF content, x_F_	0	0.2	0.5	0.7	1.0	g/g
*WPR =* 10 L/kg						
Cooking energy, *E_S_*	1.81 ± 0.01 ^a^	2.32 ± 0.03 ^b^	2.02 ± 0.05 ^b^	1.97 ± 0.24 ^a,b^	1.29 ± 0.04 ^c^	kWh/kg
Perc. of pasta water, *η_PW_*	84.2 ± 0.5 ^a^	82.3 ± 0.2 ^a^	84.7 ± 0.6 ^a^	85.0 ± 2.9 b	89.1 ± 0.1 ^c^	%
Perc. of water absorbed, *η_PC_*	7.4 ± 0.2 ^a^	7.0 ± 0.2 ^a^	6.8 ± 0.2 ^a^	6.7 ± 0.7 ^a^	8.0 ± 0.2 ^b^	%
Perc. of water evaporated, *η_EV_*	8.5 ± 0.3 ^a^	10.7 ± 0.0 ^a^	8.5 ± 0.4 ^a^	8.3 ± 2.2 ^a^	2.8 ± 0.2 ^b^	%
*WPR =* 3 L/kg						
Cooking energy, *E_S_*	1.10 ± 0.06 ^a^	1.12 ± 0.08 ^a^	1.00 ± 0.03 ^a^	1.10 ± 0.04 ^a^	0.58 ± 0.01 ^b^	kWh/kg
Perc. of pasta water, *η_PW_*	45 ± 6 ^a^	43 ± 8 ^a^	48 ± 4 ^a^	51 ± 9 ^a^	60 ± 2 ^b^	%
Perc. of water absorbed, *η_PC_*	25 ± 2 ^a^	25 ± 4 ^a^	23 ± 3 ^a^	25 ± 1 ^a^	24 ± 1 ^a^	%
Perc. of water evaporated, *η_EV_*	30 ± 4 ^a^	32 ± 4 ^a^	29 ± 2 ^a^	24 ± 7 ^a^	16 ± 1 ^b^	%

Different lowercase letters indicate statistically significant differences among the parameter means of the fresh pasta samples differing in x_F_ at the probability level of 0.05.

**Table 5:** Modeling of the simulated starch digestion process of the target product (white bread) and a few fresh pasta samples containing different fractions (x_F_) of a high-amylose wheat flour via the exponential and Peleg models, as well as estimation of the areas (AUC) enclosed by the corresponding digestograms for a digestion time of 180 min by using the above mathematical models and trapezoidal rule, starch hydrolysis index (SHI) and in vitro glycemic index (GI).

Parameter	White Bread	Fresh Pasta Enriched with High-Amylose Wheat Flour					Unit
x_F_	0	0	0.2	0.5	0.7	1.0	g/g
*C_G0_*	0.13 ± 0.03	0.053 ± 0.02	0.019 ± 0.008	0.005 ± 0.007	0.021 ± 0.021	~0	g/L
*Exponential model*							
*C_G__∞_*	0.518	0.50	0.41	0.36	0.38	0.34	g/L
*k*	0.0145 ±0.0013 ^a^	0.0087 ±0.0004 ^b^	0.0094 ±0.0007 ^b^	0.0083 ±0.0002 ^b^	0.0063 ±0.0002 ^c^	0.0048 ±0.0002 ^d^	min^−1^
R^2^	0.916	0.975	0.940	0.995	0.986	0.988	-
*AUC_E_*	68.3 ^a^	49.4 ^b^	39.8 ^c^	31.6 ^d^	29.7 ^d^	19.3 ^e^	g min/L
*GI_E_*	94	71	58	48	46	33	%
*Peleg model*							
*k*_1_	115 ± 22 ^a^	163 ± 10 ^b^	129 ± 41 ^a,b^	232 ± 13 ^c^	265 ± 22 ^c^	371 ± 37 ^d^	L min/g
*k*_2_	1.9 ± 0.3 ^a^	1.9 ± 0.1 ^a^	2.5 ± 0.2 ^b^	2.3 ± 0.1 ^b^	2.5 ± 0.1 ^b^	2.7 ± 0.2 ^b^	L/g
*R* ^2^	0.88	0.99	0.94	0.99	0.97	0.96	-
*AUC_Pele_*	74.5 ^a^	53.2 ^b^	44.9 ^b^	34.1 ^d^	33.7 ^d^	22.9 ^e^	g min/L
*GI_Peleg_*	94	70	60	48	47	35	%
*Trapezoidal Rule*							
*AUC_Trap_*	77 ± 7 ^a^	51 ± 2 ^b^	46 ± 3 ^b^	34 ± 2 ^c^	32 ± 6 ^c,d^	27 ± 1 ^d^	g min/L
*SHI*	100	66 ± 2 ^a^	59 ± 3 ^b^	44 ± 2 ^c^	42 ± 8 ^c^	36 ± 1 ^c^	%
*GI*	94	65 ± 2 a	59 ± 3 b	46 ± 2 c	44 ± 7 c	39 ± 1 c	%

Different lowercase letters indicate statistically significant differences among the parameter means of the fresh pasta samples differing in x_F_ at the probability level of 0.05.

## Data Availability

Not applicable.
